# Evidence for Retromutagenesis as a Mechanism for Adaptive Mutation in *Escherichia coli*

**DOI:** 10.1371/journal.pgen.1005477

**Published:** 2015-08-25

**Authors:** Jordan Morreall, Alice Kim, Yuan Liu, Natalya Degtyareva, Bernard Weiss, Paul W. Doetsch

**Affiliations:** 1 Department of Biochemistry, Emory University School of Medicine, Atlanta, Georgia, United States of America; 2 Graduate Program in Genetics and Molecular Biology, Emory University School of Medicine, Atlanta, Georgia, United States of America; 3 Department of Biostatistics and Bioinformatics, Rollins School of Public Health, Emory University School of Medicine, Atlanta, Georgia, United States of America; 4 Department of Pathology, Emory University School of Medicine, Atlanta, Georgia, United States of America; 5 Emory Winship Cancer Institute, Emory University School of Medicine, Atlanta, Georgia, United States of America; 6 Department of Radiation Oncology, Emory University of School of Medicine, Atlanta, Georgia, United States of America; 7 Hematology and Medical Oncology, Emory University School of Medicine, Atlanta, Georgia, United States of America; A*STAR, SINGAPORE

## Abstract

Adaptive mutation refers to the continuous outgrowth of new mutants from a non-dividing cell population during selection, in apparent violation of the neo-Darwinian principle that mutation precedes selection. One explanation is that of retromutagenesis, in which a DNA lesion causes a transcriptional mutation that yields a mutant protein, allowing escape from selection. This enables a round of DNA replication that establishes heritability. Because the model requires that gene expression precedes DNA replication, it predicts that during selection, new mutants will arise from damage only to the transcribed DNA strand. As a test, we used a *lacZ* amber mutant of *Escherichia coli* that can revert by nitrous acid-induced deamination of adenine residues on either strand of the TAG stop codon, each causing different DNA mutations. When stationary-phase, mutagenized cells were grown in rich broth before being plated on lactose-selective media, only non-transcribed strand mutations appeared in the revertants. This result was consistent with the known high sensitivity to deamination of the single-stranded DNA in a transcription bubble, and it provided an important control because it demonstrated that the genetic system we would use to detect transcribed-strand mutations could also detect a bias toward the non-transcribed strand. When residual *lacZ* transcription was blocked beforehand by catabolite repression, both strands were mutated about equally, but if revertants were selected immediately after nitrous acid exposure, transcribed-strand mutations predominated among the revertants, implicating retromutagenesis as the mechanism. This result was not affected by gene orientation. Retromutagenesis is apt to be a universal method of evolutionary adaptation, which enables the emergence of new mutants from mutations acquired during counterselection rather than beforehand, and it may have roles in processes as diverse as the development of antibiotic resistance and neoplasia.

## Author Summary

The basic principle of neo-Darwinian genetics is that mutations occurring during growth enable the subsequent survival of the mutants under selective environmental conditions. However, new mutants can arise from a non-growing bacterial population during selection in an apparently Lamarckian way. The phenomenon is called adaptive mutation. In one suggested pathway, retromutagenesis, a damaged gene produces a mutant protein that enables enough growth for a mutant gene to be copied onto daughter chromosomes. This hypothesis is supported by evidence that, in several experimental systems, a damaged gene can produce a mutant protein rather than no protein at all, and that both RNA and DNA polymerase will pair the same base with a lesion. Because this model requires gene expression before DNA synthesis, a third feature is predicted: in a non-growing population, adaptive mutations will occur preferentially on the transcribed strand of a gene. In this paper, we describe a bacterial genetic system that can distinguish between mutations occurring on either DNA strand, and we use it to confirm this prediction. The findings enhance our general understanding of evolution in all organisms, the majority of which are in a non-growing state most of the time.

## Introduction

A central tenet of genetics is that mutation precedes selection rather than the reverse. This principle was verified by isolation, through sibling selection, of mutants from cultures that had never been exposed to the growth pressure of a selective agent [1,2]. In apparent contradiction to this principle, it was later observed that new mutants would arise continuously from an ostensibly non-growing population of cells for many days after being plated on a selective medium [3–5]. The phenomenon has been termed “adaptive mutation” or “stationary-phase mutagenesis.” Much of our knowledge of the underlying mechanisms has come from studies of reversions of frameshift mutations in the *lacZ* (β-galactosidase) gene of *Escherichia coli* in a system refined by Cairns and Foster [6]. As reviewed by Roth [7], the mechanisms include: occult growth of a sub-population aided by the residual activity of the defective gene product and amplification of the gene by homologous recombination, both of which increase the number of mutational targets, and stress-related amplification, and possibly induction of *dinB*, which encodes an error-prone DNA polymerase that may produce mutations during gene amplification. Thus, the apparent Lamarckian adaptations are actually due to occult mechanisms that still conform to the neo-Darwinian principle of mutation preceding selection. Controversy still exists around the contributions of residual growth and enhanced mutation rates, and so adaptive mutagenesis is best defined simply as the process by which new mutations arise under selective conditions regardless of the mechanism [7].

Davis [8] proposed a unique mechanism to explain reversion of *lacZ* mutants in a non-growing population. The lactose in the selection medium induces transcription of the *lac* operon, and the non-transcribed strand (NTS) is displaced by mRNA in a transcription bubble. Because the NTS is now single-stranded, it is unusually susceptible to environmental damage. Before the reversion mutation specified by the DNA damage can be expressed, however, a round of DNA replication is required to copy the defect onto the transcribed strand (TS). The energy for this DNA synthesis was proposed to come from the breakdown of cellular constituents, particularly ribosomes, which is expected to occur after three days of incubation on growth media. Davis’s model predicts that the new mutations will arise from the NTS.

**Fig 1:**
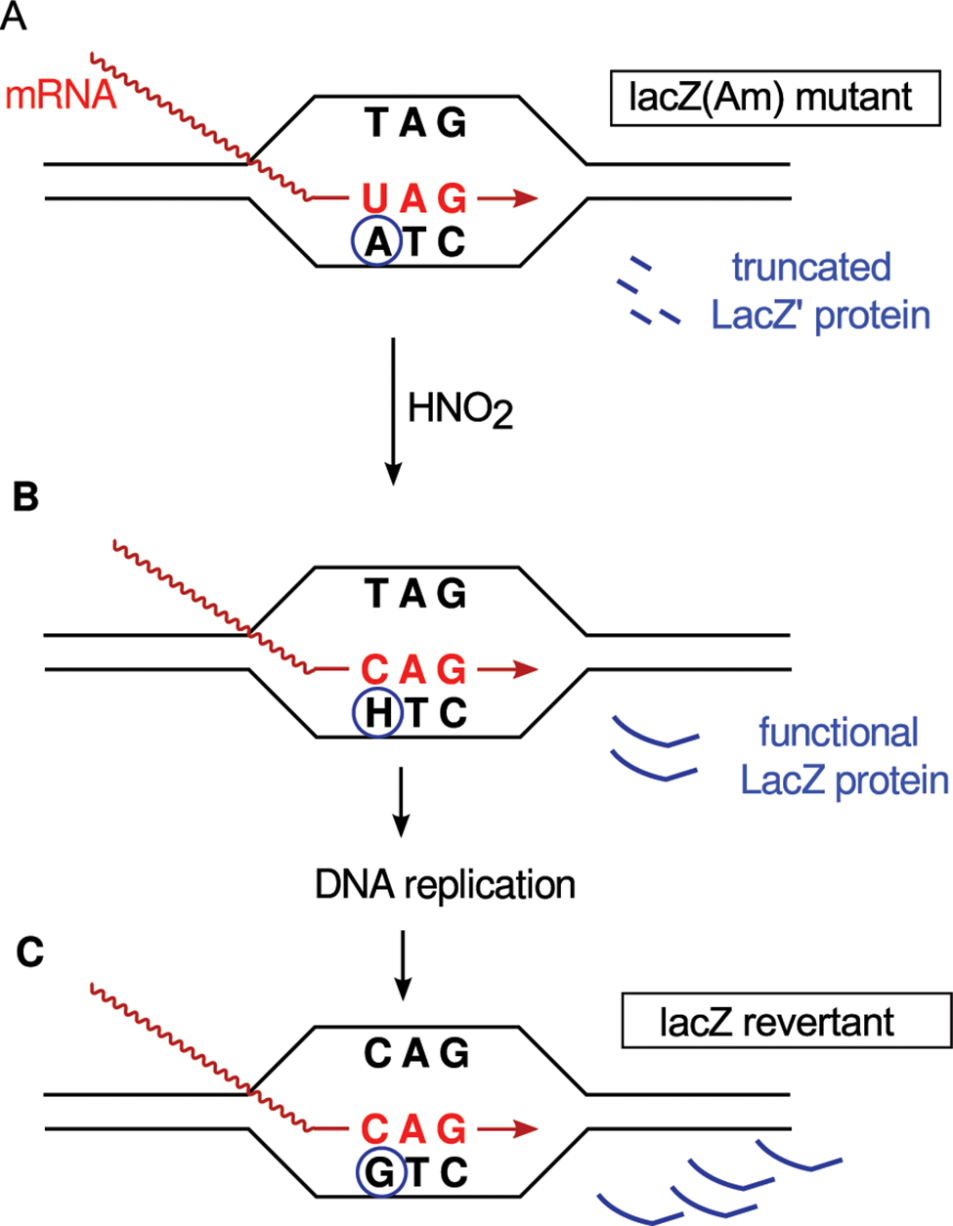
Example of retromutagenesis. Shown are schematic representations of a transcription bubble that contains an amber stop codon from which is produced inactive, truncated LacZ' polypeptide. (**A**) An adenine in the TS is deaminated by HNO_2_ to hypoxanthine (H). (**B**) RNA polymerase pairs the hypoxanthine with cytidine instead of the original uracil, leading to the translation of a full-length functional β-galactosidase (*LacZ*), which allows the cell to grow on selective media. (**C**) This gene expression enables DNA replication, which establishes the A:T → G:C mutation producing the Lac^+^ phenotype.

An alternative mechanism, which we explore in this paper, was proposed by Bridges [3] and has come to be known as “retromutagenesis” [9]. It differs from most others in that expression of a damaged gene precedes its replication. Therefore, it will only yield mutants from damage to the TS of a gene. If the DNA damage escapes repair before DNA replication, the altered gene may be transcribed and translated to produce a mutant protein that enables the cell to undergo at least one round of DNA replication during selection (Fig 1). If the DNA polymerase then misreads the lesion in the same way that the RNA polymerase did, the mutation will be permanently established in a daughter cell. In contrast, lesions on the NTS will not enable the outgrowth of mutants under stringent selection because, before the mutant gene can be expressed, the mutation specified by the lesion must be copied onto the TS by a round of DNA replication.

The first requirement for the retromutagenesis model is that a damaged gene must produce an altered protein in the absence of DNA synthesis, a process called transcriptional mutagenesis. A second requirement for retromutagenesis is that the RNA and DNA polymerases of a cell incorporate analogous nucleoside triphosphates opposite a lesion. As reviewed by Bregeon and Doetsch [10], these requirements have been fulfilled for several types of DNA lesions in both prokaryotes and eukaryotes. However, for the model to be verified, it must be demonstrated that adaptive mutation favors the TS of a gene. Two reported findings are consistent with this prediction. During selection for tryptophan prototrophy in strains with the *trp5*-A149C missense mutation, an *ogg1* mutant of *Saccharomyces cerevisiae*, defective in the repair 8-oxoguanine oxidative lesions in DNA, displayed a high frequency of late-arising Trp^+^ revertants resulting from G:C → T:A transversions [11]. The results were consistent with an implied mispairing of an 8-oxoguanine in the TS. In another experiment, 8-oxo-dGTP that was electroporated into a *trp5* mutant could cause reversions when incorporated into the NTS, but such revertants were only found after replication, and not after immediate selection under non-growth conditions [12]. Unfortunately, these studies could not test both TS and NTS strand mutations in the same experimental system.

In this paper, we describe an experimental system in *Escherichia coli* that can distinguish between mutations arising from damage to either DNA strand. We use it to test the hypothesis that retromutagenesis plays a role in the appearance of new bacterial mutants during stationary phase, and we discuss its implications for higher organisms.

## Results

### Rationale and experimental design

**Fig 2:**
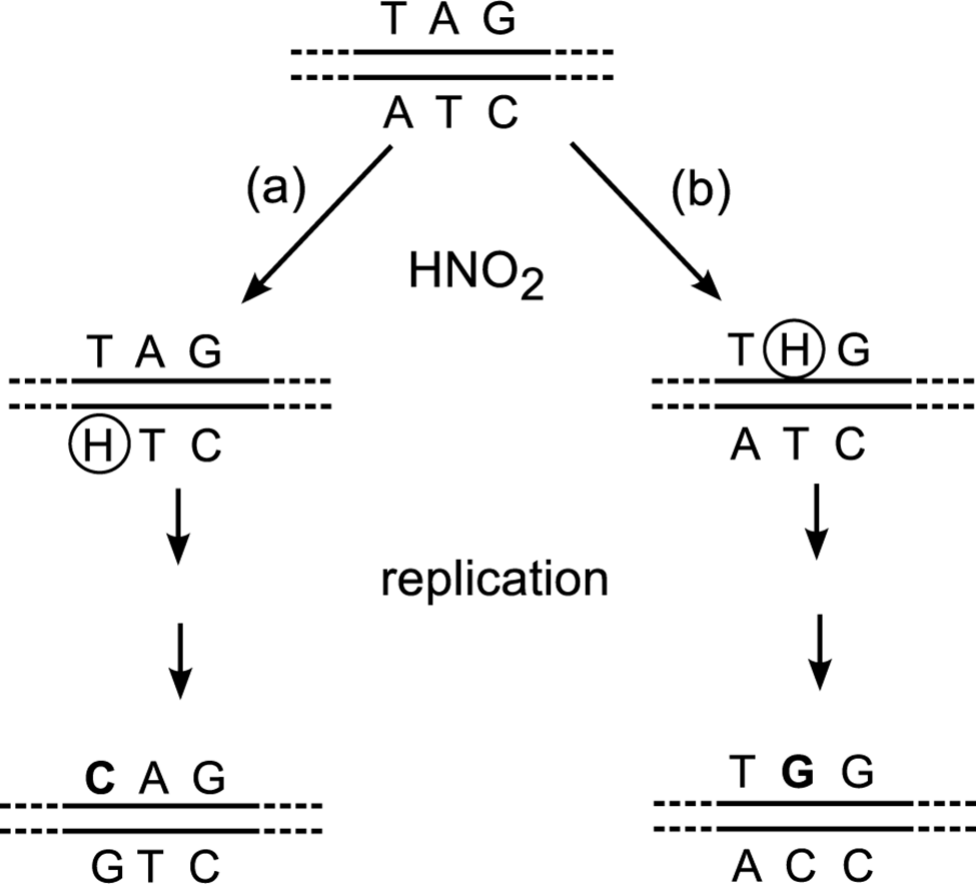
Different sequences produced by nitrosative deamination of adenine on the (a) TS or (b) NTS. Although TGG (Trp) is the wild type codon and CAG (Gln) is a missense codon, they both restore functionality (see Results), and can be distinguished through DNA sequencing of Lac^+^ revertants. H, hypoxanthine.

We have constructed an experimental system by which mutations on both strands can be separately measured. We used nitrous acid (HNO_2_), a deaminating agent that attacks adenine in an A:T base pair. The resulting hypoxanthine [13] pairs with cytosine and produces A:T→G:C transition mutations after replication. Our target was a premature TAG (amber) stop codon that replaced codon 17 (TGG) of the *lacZ* (ß-galactosidase) gene (GenBank accession number V00296.1; http://www.ncbi.nlm.nih.gov/nuccore/NC_000913.3). The amber mutants were Lac^-^, that is, unable to grow on lactose as the sole carbon source, but they could undergo HNO_2_-induced reversion to Lac^+^. The amber codon contains adenines on both the transcribed and the non-transcribed strands. From the DNA sequence of each Lac^+^ revertant, we can determine which strand was mutated (Fig 2).

The tester strains contained a disrupted allele of *nfi*, which encodes endonuclease V [14,15]. Endonuclease V catalyzes the first step in the excision repair of hypoxanthine-containing DNA [16], and *nfi* mutants have an enhanced frequency of HNO_2_-induced A:T→G:C transition mutations [17]. The tester strains also contained a chromosomal deletion for the *lac* operon. The *lacZ* amber mutant alleles were on an *attλ* element [18], which is a non-replicating DNA segment inserted at the bacterial attachment site for bacteriophage λ. Because gene orientation affects strand bias for mutagenesis, two tester strains were constructed such that the *attλ* elements (and therefore the *lacZ* genes) were in opposite orientations: the transcribed strand of *lacZ* was either the leading or the lagging strand during DNA replication.

Steps were taken to minimize DNA replication during selection because it would reduce any strand bias. The mutagenesis was performed on stationary phase cells that were starved by being washed and incubated in a nutrient-free solution, and the agar selection medium was purged of carbon sources (other than lactose) by prior growth of a Δ*lac* mutant (BW5660) on its surface. In addition, we eliminated the possibility of a sampling bias based on colony size. After 48 h of growth on lactose-minimal media the mean colony diameters (± 1 SD) were the same [1.1 (± 0.1) mm] for the both the TGG and CAG revertants, based on random samples of fifty colonies each.

### HNO_2_ treatment and outgrowth of mutants

A preliminary experiment was performed to determine whether the transcribed and non-transcribed strands were equally susceptible to damage by HNO_2_. A culture that was grown to saturation without added glucose was exposed to HNO_2_. Before being plated on lactose-minimal media, the cells were grown overnight in LB broth to allow the propagation of mutants containing lesions in either strand. Lac reversion was induced greater than 20-fold among cells treated with HNO_2_. Thirty Lac^+^ revertants were picked, all of which had NTS mutations. However, for our planned experiment to work, we needed conditions under which both strands were equally damaged at the outset so that we would see if selection produced a strand bias in the mutations. The skewed result might be explained by the presence of *lacZ* transcription bubbles at the time of HNO_2_ treatment, coupled with the suggestion by Davis [8] that the single-stranded region of an NTS in a transcription bubble is highly sensitive to DNA-damaging agents. If so, we should be able to eliminate this effect by reducing *lac* transcription through catabolite repression [19]. The *lac* operon is positively regulated by a cAMP/CAP (catabolite activator protein) complex that binds near the *lac* promoter. In the presence of glucose, the preferred carbon source, cAMP levels fall, and *lac* transcription is drastically reduced even in the presence of an inducer [19]. As predicted by our hypothesis, when we grew the cells to saturation in glucose-containing medium before mutagenesis, we no longer observed a preponderance of NTS mutations, but rather distribution of mutations balanced between the two strands, as will be shown below. This result confirmed that the strong bias toward NTS mutations that we had previously observed (when cells were grown without gluscose) was indeed due to transcription-associated mutagenesis, and it implied that DNA-bound transcripts persist in stationary phase (see Discussion). Therefore, in all subsequent experiments we grew the cells in the presence of glucose before mutagenesis to enable catabolite repression of *lac* transcription.

**Fig 3:**
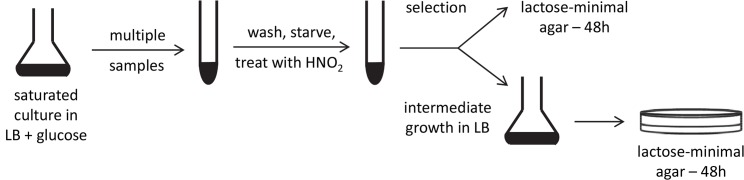
Experimental scheme. A stationary phase, saturated culture of lac mutants in LB broth + glucose was divided into many samples, washed by centrifigation, starved by incubation in 10 mM MgSO_4_, resuspended in acidic buffer, and incubated with or without added NaNO_2_. Each sample was divided in half. One part was grown in LB broth before being plated on lactose-minimal agar. The other was plated directly on the lactose-minimal agar. After 48 h, Lac^+^ revertant colonies were picked for DNA sequencing.

The strand specificity for mutagenesis was determined as detailed under Methods and outlined in Fig 3. Briefly, the amber mutant tester strains were grown to saturation with glucose, washed, and starved. Multiple samples were treated with NaNO_2_ in an acidic buffer, with a survival of 29 to 33%. After the treatment was stopped with a neutral buffer, each sample was divided in two. One portion was spread immediately on the selective medium; the other was grown in LB broth before selection (Fig 3). If reversion occurred entirely by retromutagenesis, direct plating would reveal that only TS mutations were selected, whereas after intermediate growth, both TS and NTS mutations would appear among the revertants.

Exposure to HNO_2_ increased the frequency of Lac^+^ revertants by 18- to 51-fold (Table 1). Because of the relatively low frequency of spontaneous mutants and their statistically small sample size, the sequencing data for the HNO_2_-induced revertants (Table 2) were not corrected for those of the untreated samples. When the mutagenized cultures were allowed to grow in LB broth before selection, they yielded CAG and TGG Lac^+^ revertants in almost equal number (Table 2), indicating that both strands were about equally mutagenized (*p* = 0.7, Fisher exact test). However, when the mutagenized cells were plated directly on selective medium, without intermediate growth, CAG mutants predominated, indicating a preference for TS mutations (*p* < 0.01, Fisher's exact test) in strains with either gene orientation. Therefore, during selection, most mutations of the NTS were not replicated, allowing those of the TS to prevail. The results were not significantly affected by gene orientation (Table 2) (*p* < 0.01, Cochran-Mantel-Haenszel test), which is consistent with our expectation that at the time of treatment with HNO_2_, stationary phase cells did not contain a significant number of replication forks. These findings support the transcription-mediated mechanism of retromutagenesis for the inheritance of new mutations under selective conditions.

**Table 1:** Reversion frequencies after exposure to nitrous acid.

Gene orientation^a^ and strain	Lac^+^ selection	HNO_2_ exposure	Lac^+^ revertants per 10^8^ viable cells	HNO_2_-induced/spontaneous revertants
Leading strand (strain Z126)	Immediate	-	0.9	33
		+	30	
	After non-selective growth	-	1.3	38
		+	50	
Lagging strand (strain Z127)	Immediate	-	1.1	18
		+	20	
	After non-selective growth	-	1.0	51
		+	51	

a) Gene orientation refers to the directionality of the transcribed strand at the replication fork.

**Table 2:** Spectrum of mutations in a *lacZ* amber codon after HNO_2_ mutagenesis.

Gene orientation^a^ (strain)	Lac^+^ selection	Lac^+^ revertants^b^		
		CAG (TS)	TGG (NTS)	Other^c^
Leading strand (Z126)	Immediate	47	6	6
	After growth	22	23	9
Lagging strand (Z127)	Immediate	49	5	7
	After growth	21	24	9

a) Gene orientation refers to the directionality of the transcribed strand at the replication fork.

b) For each strain, Lac^+^ revertants were picked from 14 batches of cells that were separately treated with HNO_2_. For cells that were plated after overnight growth in broth, one revertant colony from each of 56 independent cultures was analyzed (see Methods).

c) Other mutations consisted of in-codon transversions and uncharacterized suppressor mutations (see S3 Table).

## Discussion

Retromutagenesis provides a simple, direct explanation for adaptive mutation. However, it is not as obvious as it first seems because it relies on several tacit assumptions. Retromutagenesis requires that RNA polymerases bypass lesions in DNA and incorporate mutations analogous to those caused by DNA polymerase bypass (described in **Introduction**). Our experimental demonstration relied on a further assumption, which we verified. During selection, the starved cells had to be able to harvest enough energy to make functional protein (β-galactosidase) from the damaged gene before they have enough energy for DNA synthesis. In addition, our results provide evidence, albeit indirect, that hypoxanthine can be added to the list of abnormal bases [10] that undergo similar base mispairing when bypassed by either DNA or RNA polymerase in *E*. *coli*.

For many years, adaptive mutation has been operationally defined by the observation of mutagenesis during selection as evidenced by the continuous outgrowth of new mutants many days after selective plating, during which there is no observable background growth of the parental cells. In our experiments, however, DNA damage was induced before selection, and the mutant colonies appeared by 48 h of growth. The rapid, exclusive growth of revertants under selection enabled us to avoid interference from growth-dependent mechanisms that may contribute to adaptive mutation. Most of the previous experiments on adaptive mutation have attempted to rule out growth of the parent cells during selection, which is almost impossible to do with the necessary rigor. Although there may be no observable overall growth, there might still be growth of a sub-population that gives rise to new mutants. Consequently, it is generally acknowledged that much apparent adaptive mutation stems from occult growth of at least some parental cells, or at least of parts of their chromosomes through gene amplification [7]. In our approach, however, there is no need to rule out DNA replication occurring before selection, because it would only have reduced the observed strand bias. Instead of measuring overall DNA replication via cellular growth in the parental population during selection as in previously published experiments, we are able to gauge DNA replication specifically in the revertants via NTS mutations. Low frequency of NTS mutations found after direct selection indicates there was little DNA synthesis before gene expression and strongly supports the model of retromutagenesis.

Our studies employed an amber mutation in the tester strains. The expected low residual activity of the protein containing the amber mutation minimized growth of the parental cells during selection. Its position in a dispensable part of the *lacZ* gene [20] meant that unlike most other mutation indicators, it should be able to revert by more than one type of amino acid substitution, enabling detection of mutations at different positions in the codon. However, there is a caveat to using this method as a general approach. Although the early region of the gene can be deleted without significant loss of function, it is possible that some amino acid substitutions may cause a deleterious alteration of protein structure. In a preliminary experiment, we found that we were unable to use an amber mutation in codon 3 of *lacZ*. Following mutagenesis and intermediate growth, rapidly growing revertants displayed AT → GC mutations at only the first position of the codon, which restored the wild type sequence. Mutants with transitions at the second position of the codon grew too slowly on the selection plates.

When the cells were grown without added glucose, exposed to HNO_2_, and then grown before selection, all of the revertants studied were NTS mutants. Although transcription-coupled repair [21] could create such a strand bias by selectively repairing the TS, it acts only on lesions that obstruct RNA polymerase and that are repaired by the UvrABC system, both of which are not features expected of hypoxanthine in DNA. Our subsequent demonstration of a high frequency of TS mutations during both direct and delayed selection after catabolite repression (Table 2) provides direct experimental evidence that transcription-coupled repair is not functioning at a high level on these lesions. NTS mutations in the absence of catabolite repression are best explained by “transcription-associated mutagenesis” (not to be confused with “transcriptional mutagenesis”), reviewed by Jinks-Robertson and Bhagwat [22]. This mechanism provides the basis for an alternative model of adaptive mutation, proposed by Davis [8], which was prompted by the widespread observation that single-stranded DNA, such as that in a transcription bubble, is unusually sensitive to many types of DNA damage. This susceptibility has been specifically confirmed for nitrosative deamination *in vitro*, which is ten times faster in single-stranded than in double-stranded DNA [23]. In contrast to retromutagenesis, the Davis model states that selectable mutations result from damage to the NTS, and a round of DNA replication is required to encode a mutation in the TS for gene expression to begin. Our results indicated that our experimental system can detect mutational bias toward the NTS as well as toward the TS, although we did not specifically test the contribution of this transcription-associated mutagenesis to adaptive mutation. However, we may have gained some insight into its significance. The extent of this NTS bias surprised us for several reasons. First, previous reports had indicated only a fourfold to tenfold increase in spontaneous mutations of the NTS during transcription, although mutations on the TS were not studied at the same time [24,25]. Second, we did not expect persistent transcripts of the *lac* operon in our starved, stationary phase cells at the time of mutagenic treatment. Our strains had been constructed with a tested, repressible *lac* operon and they had been grown without an added inducer. The explanation may lie in the spontaneous cAMP-dependent derepression of the *lac* operon observed in cells entering the stationary phase [26] and in the possible contamination, by lactose, of tryptone (digested casein) in the medium [27]. Third, it seemed unlikely that DNA-bound RNAs should persist in stationary phase cells. They should be digested by ribonuclease HI, or else they could serve as primers for aberrant (“constitutive stable”) DNA replication [28]. It appears that stationary-phase cells may be primed for transcription-associated mutagenesis and adaptive mutation by the induction of cAMP-regulated genes and the persistence of transcription bubbles.

How widespread is retromutagenesis in nature? Although it does not apply to growing cells, most organisms in nature and most cells in our body exist in a state of limited growth. Retromutagenesis applies only to dominant mutations, but this should be true of most adaptive mutations, which are usually due to a gain of function. Theoretically, retromutagenesis cannot provide resistance to inhibitors of transcription or of protein synthesis because it relies on gene expression to enable the initial DNA synthesis that establishes heritability. It could, however, mediate resistance to DNA synthesis inhibitors or to tumor suppressors. For example, adaptive mutation to ciprofloxacin resistance was observed in *E*. *coli*; new mutants arose continuously days after plating in the presence of the antibiotic [29]. During this time there was no measurable growth of the parental cells; ciprofloxacin is an inhibitor of gyrase and therefore of DNA synthesis. Remarkably, in a parallel experiment, stationary phase cells did not become resistant to rifampin, an inhibitor of RNA polymerase. Although it was not appreciated at the time, these combined findings point to retromutagenesis as the adaptive mechanism. Similarly, retromutagenesis could be involved in resistance to the antimicrobial agent trimethoprim or the antitumor compound methotrexate, both based on dominant mutations that block the binding of the drugs to dihydrofolate reductase [30,31], which would otherwise ultimately inhibit DNA synthesis. Other possible examples in eukaryotes are the common mutations in *p53* that turn this tumor suppressor gene into a dominant transforming oncogene [32] and dominant gain-of-function mutations in *JAK2* that result in myeloproliferative disorders [33]. Similarly, TM can induce single-base changes in Ras transcripts that cause pro-growth increases in phosphorylated ERK protein [34].

Retromutagenesis is a transcription-mediated process of adaptive mutation that requires a damaged gene to be expressed before it can be replicated. In light of previous evidence, it is probably one of several mechanisms by which cells can undergo directed selection in the absence of apparent growth.

## Materials and Methods

### Strain construction

The *E*. *coli* K-12 F^-^λ^-^ strains used in this study and the details of their construction are listed in S1 Table. We constructed two tester strains that had amber mutations in *lacZ* genes in opposite orientations on the chromosome. The amber mutation in codon 17 of *lacZ* was previously generated by oligonucleotide-mediated transformation. Subsequent gene transfers were by generalized transduction with phage P1 *dam rev6* [35]. To orient the genes differently, we used *attλ* elements, which were plasmid-derived DNA segments that had integrated into the bacterial chromosome in opposite directions at the attachment site of phage λ. They contained a selectable ampicillin resistance marker and a *lac* operon with a *lacZ* missense mutation. They were transduced into an amber suppressor strain, BW5660, to create strains Z122 and Z123. Then the *lacZ* amber allele was transduced into *attλ* elements of these strains, crossing out the recipient's *lac* missense mutation. (The chromosomal *lac* deletions encompassed a genomic region too large to undergo P1-mediated transduction.) Selection was for the Lac^+^ phenotype specified by the suppressed *lacZ* amber allele. The *lacI* (constitutive) mutations in the recipients were crossed out at the same time, as determined by scoring with X-Gal and IPTG [36]. Finally, the *attλ* elements, which specified ampicillin resistance, were transduced into BW1181, a non-suppressing strain that had an *nfi* mutation and a *lac* deletion, thereby creating strains Z126 and Z127, which had the following relevant genotype: Δ*lac nfi-1*::*cat attλ*::[*lacZ*(Am)*Y* ^*+*^ *Z* ^*+*^]. In strain Z126, the *lacZ* gene on the *attλ* element is codirectional with the leading strand during DNA synthesis, and in strain Z127, it is in the opposite orientation. The *lac* point mutations were confirmed by DNA sequencing. The *nfi-1*::*cat* insertion and the orientations of the *attλ* elements were confirmed by PCR with primers listed in S2 Table.

### Bacteriological media

LB media were the rich media used used for routine growth. Lactose-minimal agar was Medium E [37] supplemented with 0.1% lactose (Sigma Aldrich, > 99% pure by gas chromatography) and thiamine at 1 μg/ml. It was pretreated with a Δ*lac* scavenger strain, BW5660 (S1 Table), to remove traces of carbon sources other than lactose [38]: a saturated culture of strain BW5660 grown in LB broth was centrifuged and resuspended in one-tenth its original volume of 10 mM MgSO_4_, and 0.1 ml was spread on each plate and incubated for 48 h at 37°C.

### Mutagenesis

Strains Z126 and Z127 were grown overnight with aeration at 37°C in LB broth containing 0.4% glucose. The inocula contained less than 10^4^ cells so that they would be unlikely to contain spontaneous revertants. The saturated cultures were centrifuged, and the cells were washed and resuspended in 10 mM MgSO_4_, then starved by incubation with shaking for 30 min at 37°C. They were separated into twenty-eight 1-ml aliquots and centrifuged. The cells were resuspended in 0.5 ml of 0.1 M sodium acetate buffer (pH 4.6) with or without 80 mM NaNO_2_ and incubated for 9 min at 37°C. The reactions were stopped by the addition of 5.0 ml of cold Medium A buffer [36]. The remaining cells were centrifuged and resuspended in 0.4 ml of 10 mM MgSO_4_. From each treated sample, 0.2 ml were spread on lactose-minimal agar (14 plates), and 0.05 ml were added to each of 4 tubes (56 total) containing 1.8 ml of LB broth and incubated overnight with shaking at 37°C. The untreated samples were handled similarly, except that the intermediate growth was performed in two tubes each. The saturated cultures were centrifuged, and the cells were resuspended in 10 mM MgSO_4_ and plated on lactose-minimal media. Before and after overnight growth, two samples each of the treated and untreated cells were diluted and plated on LB agar to determine cell survival and cell concentration. After 48 h of growth at 37°C, random, uniformly large Lac^+^ colonies were picked for DNA sequencing.

### Other methods

Colony diameters were measured with a loupe containing a reticle (Edmund Scientific Co.). The *5'-*terminal *lacZ* region of each Lac^+^ colony to be sequenced was amplified by colony PCR [39] with primers lacZ-249F and lacZ+178R (S2 Table). Sequencing was carried out by Beckman-Coulter Genomics (Danvers, MA) via dye termination capillary electrophoresis on an ABI 3730XL (Applied Biosystems, Inc.) instrument, using the lacZ-249F primer. Statistical significances were determined by the two-tailed Fisher's exact test [40] using an online interface (http://www.langsrud.com/fisher.htm), and the Cochran–Mantel–Haenszel test [41] using a published spreadsheet (http://www.biostathandbook.com/cmh.xls).

## Supporting Information

S1 Table Bacterial strains used. a) All strains were derivatives of *E*. *coli* K-12 F^-^λ^-^. b) The *attλ* insertion elements contain the entire *lac* operon, with mutations in the *lacI* and *lacZ* genes where indicated, and a functional β-lactamase (*bla*) gene specifying ampicillin resistance (Amp^r^). “Left” and “Right” refer to the orientation of the *lac* operon in the *attλ* elements with respect to the origin of DNA replication. The *lacZ*(*CC106*) allele has as missense mutation at nucleotide (nt) 1384. Non-standard abbreviations: Am, amber mutation; AS, amber suppressor; nt, nucleotide. c) Transductions with phage P1 are described as follows: P1(donor) × recipient → selected phenotype. Amp^r^, ampicillin resistance.
(DOCX)

S2 Table Oligonucleotide primers used. (DOCX)

S3 Table Spectrum of unanticipated sequences in a *lacZ* ^+^ revertants at amber codon after nitrous acid mutagenesis^a^. a) The table shows the breakdown of revertants in the category “Other” in Table 2 of Results. b) Gene orientation refers to the directionality of the transcribed strand at the replication fork. c) Revertants that retained the original TAG codon had unmapped suppressor mutations, by definition. The other mutations are transversions, the frequencies of which are enhanced by HNO_2_ in both wild type and *nfi* mutant cells [17]. TAA, which should have resulted from the deamination of guanine or cytosine at the third position of the codon [17], was not found in the revertants because it is a stop codon.
(DOCX)
