# Supplementary material for: Evidence for Retromutagenesis as a Mechanism for Adaptive Mutation in Escherichia coli
Source: PLoS Genet. 2015 Aug 25;11(8):e1005477. doi: 10.1371/journal.pgen.1005477 (PMC4548950; doi:10.1371/journal.pgen.1005477)
Supplement: S1 Table — a) All strains were derivatives of E. coli K-12 F-λ-. b) The attλ insertion elements contain the entire lac operon, with mutations in the lacI and lacZ genes where indicated, and a functional β-lactamase (bla) gene specifying ampicillin resistance (Ampr). “Left” and “Right” refer to the orientation of the lac operon in the attλ elements with respect to the origin of DNA replication. The lacZ(CC106) allele has as missense mutation at nucleotide (nt) 1384. Non-standard abbreviations: Am, amber mutation; AS, amber suppressor; nt, nucleotide. c) Transductions with phage P1 are described as follows: P1(donor) × recipient → selected phenotype. Ampr, ampicillin resistance. (DOCX) [file pgen.1005477.s001.docx]

**Supplementary Table 1: Bacterial strains used**

| **Strain^a^** | **Relevant genotype^b^** | **Reference or source^c^** |
| --- | --- | --- |
| BW1181 | *nfi-1*::*cat* Δ(*argF-lac*)*169* | B. Weiss |
| BW1948 | Δ(*cro-bioA*) *lacZ*(Am) (nt 50, with G→A) | Suppl. Ref 1 |
| BW5660 | Δ(*gpt-lac*)5 *glnX44*(AS) | Suppl. Ref 1 |
| EC3150 | *att*λ::[*lacI lacZ*(*CC106*) *bla*]Left | Suppl. Ref 2 |
| EC3209 | *att*λ::[*lacI lacZ*(*CC106*) *bla*]Right | Suppl. Ref 2 |
| Z122 | *attλ*::[*lacI lacZ(CC106) bla*]Right Δ(*gpt-lac*)5 *glnX44*(AS) | P1(EC3209) × BW5660 → Amp^r^ |
| Z123 | *att*λ::[*lacI lacZ*(*CC106*) *bla*]Left Δ(*gpt-lac*)5 *glnX44*(AS) | P1(EC3150) × BW5660 → Amp^r^ |
| Z124 | *att*λ::[*lacZ*(Am)(nt 50, G→A) *bla*]Right Δ(*gpt-lac*)5 *glnX44*(AS) | P1(BW1948) × Z122 → Lac^+^, inducible |
| Z125 | *att*λ::[*lacZ*(Am)(nt 50, G→A) *bla*]Left Δ(*gpt-lac*)5 *glnX44*(AS) | P1(BW1948) × Z123 → Lac^+^, inducible |
| Z126 | *att*λ::[*lacZ*(Am) (nt 50, G→A) *bla*]Right Δ(*argF-lac*)*169 nfi-1*::*cat* | P1(Z124) × BW1181 → Amp^r^ |
| Z127 | *att*λ::[*lacZ*(Am) (nt 50, G→A) *bla*]HLeft Δ(*argF-lac*)*169 nfi-1*::*cat* | P1(Z125) × BW1181 → Amp^r^ |

Supplementary reference

1. Wanner BL (1986) Novel regulatory mutants of the phosphate regulon in Escherichia coli K-12. J Mol Biol. Sep 5;191(1):39-58. PMID: 3540312

2. Fijalkowska IJ, Jonczyk P, Tkaczyk MM, Bialoskorska M, Schaaper RM (1998) Unequal fidelity of leading strand and lagging strand DNA replication on the Escherichia coli chromosome. Proc Natl Acad Sci U S A. Aug 18;95(17):10020-5. PMID: 9707593
