# Supplementary material for: Evidence for Retromutagenesis as a Mechanism for Adaptive Mutation in Escherichia coli
Source: PLoS Genet. 2015 Aug 25;11(8):e1005477. doi: 10.1371/journal.pgen.1005477 (PMC4548950; doi:10.1371/journal.pgen.1005477)
Supplement: S2 Table — (DOCX) [file pgen.1005477.s002.docx]

**Supplementary Table 2. Oligonucleotide primers used**

| **Primers** | **Sequence (5' → 3')** | **Target** |
| --- | --- | --- |
| lacZ-249F | TCAGCTGTTGCCCGTCTCAC | *lacZ*(Am) codon 17 |
| lacZ+178R | CGCCATTCGCCATTCAGG | *lacZ*(Am) codon 17 |
| nfi::catF | ATGGATCTCGCGTCATTAC | *nfi-1*::*cat* insertion |
| nfi::catR | CAGTTTACCTGAATTAGGG | *nfi-1*::*cat* insertion |
| pLDR9 MCS 5′F | AGGTGCCTCACTGATTAAGC | *attλ* element orientation, with lac insert 5′ B |
| lac insert 5′ B | CCCGTTATAGGAGTGC | *attλ* element orientation, with pLDR9 MCS 5′F |
| PstI 5′ F | ATGGATAAGCTTGGGCTGCA | *attλ* element orientation, with PstI 5′ R |
| PstI 3′ R | GGGATCCTCTAGAGTCGACC | *attλ* element orientation, with PstI 5′ F |
