# Supplementary material for: Evidence for Retromutagenesis as a Mechanism for Adaptive Mutation in Escherichia coli
Source: PLoS Genet. 2015 Aug 25;11(8):e1005477. doi: 10.1371/journal.pgen.1005477 (PMC4548950; doi:10.1371/journal.pgen.1005477)
Supplement: S3 Table — a) The table shows the breakdown of revertants in the category “Other” in Table 2 of Results. b) Gene orientation refers to the directionality of the transcribed strand at the replication fork. c) Revertants that retained the original TAG codon had unmapped suppressor mutations, by definition. The other mutations are transversions, the frequencies of which are enhanced by HNO2 in both wild type and nfi mutant cells [17]. TAA, which should have resulted from the deamination of guanine or cytosine at the third position of the codon [17], was not found in the revertants because it is a stop codon. (DOCX) [file pgen.1005477.s003.docx]

**Supplementary Table 3. Spectrum of unanticipated sequences in a *lacZ*^+^ revertants at amber codon after nitrous acid mutagenesis^a^**

| **Gene orientation^b^** | **Lac^+^ selection** | **Lac^+^ revertants** | | | | |
| --- | --- | --- | --- | --- | --- | --- |
|  |  | **AAG** | **TAT** | **TTG** | **GAG** | **TAG^c^** |
| Leading strand (strain Z126) | Immediate | 2 | 1 | 0 | 0 | 3 |
|  | After intermediate growth | 3 | 1 | 1 | 0 | 4 |
| Lagging strand (strain Z127) | Immediate | 2 | 2 | 1 | 0 | 2 |
|  | After intermediate growth | 1 | 2 | 0 | 2 | 4 |
